# Supplementary material for: Effects of Different Yeasts on Physicochemical and Oenological Properties of Red Dragon Fruit Wine Fermented with Saccharomyces cerevisiae, Torulaspora delbrueckii and Lachancea thermotolerans
Source: Microorganisms. 2020 Feb 25;8(3):315. doi: 10.3390/microorganisms8030315 (PMC7142936; doi:10.3390/microorganisms8030315)
Supplement: Supplementary file 1 [file microorganisms-08-00315-s001.pdf]

Supplementary materials

Effects of different yeasts on physicochemical and oenological properties of red dragon fruit wine fermented with *Saccharomyces cerevisiae, Torulaspora delbrueckii* and *Lachancea thermotolerans*

Xiaohui Jiang1, Yuyun Lu1*, Shao Quan Liu1,2*

[1] Department of Food Science and Technology, National University of Singapore, Science Drive 2, Singapore 117543, Singapore
[2] National University of Singapore (Suzhou) Research Institute, 377 Lin Quan Street, Suzhou Industrial Park, Jiangsu 215123, China

*Corresponding author. Postal address: Department of Food Science and Technology, National University of Singapore, Science Drive 3, Singapore. Tel.: +65 6516 2687; fax: +65 6775 7895.
E-mail address: fstluy@nus.edu.sg (Yuyun Lu); fstlsq@nus.edu.sg (Shao-Quan Liu)

Table S1. Major volatile compounds (GC-FID peak area x 10^{6}) and their relative peak areas (RPA) identified in red dragon fruit juice and wines fermented with *Saccharomyces* and non-*Saccharomyces* yeast strains

		Day 0		Day 14						
				Biodiva[#]		Concerto[#]		EC-1118[#]		
**Volatile compounds**	**LRI[$]**	**Peak area**	**RPA%**	**Peak area**	**RPA %**	**Peak area**	**RPA%**	**Peak area**	**RPA %**	**Organoleptics[Ø]**
**Acid**										
Acetic acid	1453	1.27 ± 0.14[a]	0.93	4.33 ± 0.37[b]	0.10	5.85 ± 0.57[c]	0.15	5.20 ± 0.49[c]	0.13	Acidic, pungent, vinegar-like
Hexanoic acid	1836	20.00 ± 1.82[a]	14.60	ND[&]		ND		ND		Cheesy, fruity, fatty
Octanoic acid	2052	9.71 ± 1.27[a]	7.08	2.86 ± 0.17[b]	0.06	2.06 ± 0.23[b]	0.05	6.30 ± 0.46[c]	0.15	Acidic, cheesy, fruity
Nonanoic acid	2160	1.29 ± 0.42[a]	0.94	0.26 ± 0.01[b]	0.01	ND		ND		Waxy, cheesy, dairy
Decanoic acid	2264	ND		ND		ND		2.29 ± 0.25	0.06	Fatty, coconut,
	Subtotal	32.28	23.55	7.46	0.17	7.92	0.21	13.79	0.33	
**Alcohols**										
Ethanol	992	ND		3959.6 ± 89.9[a]	89.50	3476.0 ± 275.5[b]	91.57	3780.1 ± 105.2[a]	90.95	Alcoholic, solventy
1-Propanol	1025	ND		ND		5.63 ± 0.63[a]	0.15	10.79 ± 0.38[b]	0.26	Alcoholic, fruity, musty
Isoamyl alcohol	1229	ND		172.4 ± 9.7[a]	3.90	108.5 ± 7.7[b]	2.86	62.37 ± 3.35[c]	1.50	Alcoholic, whiskey, fruity, banana
1-Hexanol	1371	79.24 ± 5.06[a]	57.81	4.06 ± 0.12[a]	0.09	ND		ND		Fusel oily, fruity, alcoholic, sweet, green
2-Phenylethyl alcohol	2005	ND		172.2 ± 17.1[a]	3.89	98.71 ± 3.83[b]	2.60	78.74 ± 1.48[c]	1.89	Floral, rose, dried rose
	Subtotal	79.24	57.81	4308.23	97.38	3688.85	97.18	3932.04	94.61	
**Aldehydes**										
Hexanal	1085	11.60 ± 1.89[a]	8.46	ND		ND		ND		Fresh, green, grass leafy
2-Hexenal	1222	2.01 ± 0.09[a]	1.46	ND		ND		ND		Fruity, green, leafy, vegetable
Benzaldehyde	1538	ND		1.90 ± 0.14[a]	0.04	ND		ND		Almond like
Phenyl acetaldehyde	1651	2.59 ± 0.23[a]	1.89	ND		ND		ND		Floral, sweet
2,4-Dimethyl benzaldehyde	1838	ND		0.26 ± 0.01[a]	0.01	ND		ND		Cherry, almond, spicy, vanilla
	Subtotal	16.19	11.81	2.16	0.05	ND		ND		

Table S1. Continued.

		Day 0		Day 14						
				Biodiva[#]		Concerto[#]		EC-1118[#]		
Volatile compounds	LRI[$]	Peak area	RPA %	Peak area	RPA%	Peak area	RPA%	Peak area	RPA %	Organoleptics[∅]
**Ketones**										
3-Hydroxy-2-butanone	1305	ND		1.14 ± 0.08^{a}	0.03	ND		4.50 ± 0.15^{b}	0.11	Sweet, buttery, creamy, milky
Ethylidene acetone	1391	ND		0.74 ± 0.06^{a}	0.02	ND		ND		Fruity, acetone, phenolic, fishy
	Subtotal	ND		1.89	0.04	ND		4.50	0.11	
**Ester**										
Ethyl acetate	968	ND		15.88 ± 0.76^{a}	0.36	25.58 ± 1.93^{b}	0.67	15.78 ± 1.67^{a}	0.38	Ethereal, fruity, solventy
Isoamyl acetate	1115	ND		19.06 ± 1.58^{a}	0.43	9.42 ± 0.63^{b}	0.25	8.08 ± 0.7^{b}	0.19	Sweet, banana, ripe
Methyl hexanoate	1174	2.16 ± 0.23	1.58	ND		ND		ND		Fruity, pineapple, ethereal
Ethyl hexanoate	1215	ND		20.93 ± 1.64^{a}	0.00	32.54 ± 4.37^{b}	0.86	41.93 ± 1.03^{c}	1.01	Sweet, fruity, pineapple, green
Hexyl acetate	1262	ND		5.69 ± 0.58^{a}	0.13	ND		5.78 ± 0.53^{b}	0.14	Fruity, green, apple, banana, sweet
Ethyl heptanoate	1320	ND		0.52 ± 0.01^{a}	0.01	0.43 ± 0.01^{b}	0.01	0.48 ± 0.00^{c}	0.01	Fruity, pineapple, cognac, rummy, winey
Ethyl octanoate	1425	0.22 ± 0.08	0.16	16.58 ± 0.47^{a}	0.37	12.94 ± 1.32^{b}	0.34	83.55 ± 5.68^{c}	2.01	Fruity, pineapple, creamy, dairy
Ethyl nonanoate	1523	ND		ND		1.01 ± 0.12^{a}	0.03	0.60 ± 0.04^{b}	0.01	Sweet, fruity, pear
Ethyl decanoate	1627	ND		5.87 ± 0.35^{a}	0.13	2.61 ± 0.49^{b}	0.07	21.02 ± 1.45^{c}	0.51	Sweet, waxy, apple
Diethyl succinate	1670	ND		0.24 ± 0.03^{a}	0.01	0.18 ± 0.02^{b}	0.00	0.18 ± 0.01^{b}	0.00	Fruity, cooked apple, passion fruit
Ethyl 9-decenoate	1681	ND		0.28 ± 0.04^{a}	0.01	ND		0.64 ± 0.08^{b}	0.02	Fruity, fatty
Ethyl phenylacetate	1784	ND		0.21 ± 0.03^{a}	0.00	0.30 ± 0.01^{b}	0.01	0.20 ± 0.02^{a}	0.00	Sweet, floral, honey, rose, balsamic, cocoa
2-Phenethyl acetate	1816	ND		1.55 ± 0.12^{a}	0.04	0.45 ± 0.02^{b}	0.01	2.34 ± 0.17^{c}	0.06	Floral, rose, sweet, honey, fruity, tropical
Ethyl dodecanoate	1834	ND		5.88 ± 0.41^{a}	0.13	5.36 ± 0.39^{a}	0.14	12.94 ± 2.81^{b}	0.31	Sweet, waxy, fruity, apple, grape, brandy
Isobutyl pentadecanoate	1855	ND		0.34 ± 0.00^{a}	0.01	ND		0.62 ± 0.05^{b}	0.01	Fatty

Table S1. Continued.

Volatile compounds	LRI[$]	Day 0		Day 14						Organoleptics[Ø]
				Biodiva[#]		Concerto[#]		EC-1118[#]		
		Peak area	RPA %	Peak area	RPA %	Peak area	RPA %	Peak area	RPA %	
2-Phenethyl propionate	1884	ND		0.43 ± 0.08[a]	0.01	ND		ND		Floral, rose, fruity, honey, balsamic
Ethyl myristate	2042	ND		2.21 ± 0.27[a]	0.05	1.61 ± 0.07[b]	0.04	2.63 ± 0.24[a]	0.06	Sweet, waxy, violet
Ethyl palmitate	2253	ND		ND		ND		1.51 ± 0.06[a]	0.04	Waxy, fruity, creamy, milky, balsamic
	Subtotal	2.37	1.73	95.66	1.69	92.44	2.44	198.28	4.77	
**Terpene derivatives**										
β-Myrcene	1140	0.77 ± 0.09[a]	0.56	2.74 ± 0.33[b]	0.06	4.63 ± 0.52[c]	0.12	3.37 ± 0.32[d]	0.08	Peppery, terpenic, spicy, balsamic
D-Limonene	1170	ND		1.40 ± 0.09[a]	0.03	ND		ND		Citrus, orange, fresh, sweet
Linalool	1539	4.96 ± 0.41[a]	3.62	0.77 ± 0.07[b]	0.02	0.90 ± 0.04[c]	0.02	0.65 ± 0.07[b]	0.02	Citrus, floral, sweet, rose
Longifolene	1554	0.94 ± 0.07[a]	0.69	3.47 ± 0.42[b]	0.08	1.26 ± 0.04[c]	0.03	1.33 ± 0.05[c]	0.03	Sweet, woody, rose
α-Terpineol	1697	0.30 ± 0.02[a]	0.22	0.20 ± 0.03[b]	0.00	ND		ND		Pine, terpenic, lilac, citrus, woody, floral
	Subtotal	6.98	5.09	8.59	0.19	6.80	0.18	5.35	0.13	
**Phenols**										
2-Methoxy-4-vinylphenol	2211	ND		ND		ND		2.12 ± 0.15[a]	0.05	Woody, fresh, cedar, peanut
	**Total**	137.06	100.00	4423.98	100.00	3796.00	100.00	4156.08	100.00	

[$] LRI: liner retention index determined in the DB-FFAP column.

[#] Biodiva, *Torulaspora delbrueckii*; Concerto, *Lachancea thermotolerance*; EC-1118, *Saccharomyces cerevisiae.*

[Ø] Organoleptic descriptors were obtained from http://www.thegoodscentscompany.com (accessed 8 October, 2018).

[&] ND: not detected.

[a, b ,c, d] Statistical analysis ANOVA (n=3) at 95% confidence level with the same letter indicating no significant difference.
